# Health care informational challenges for women diagnosed with cervical intraepithelial neoplasia: a qualitative study

**DOI:** 10.1186/s12905-019-0811-5

**Published:** 2019-09-02

**Authors:** Carla Freijomil-Vázquez, Denise Gastaldo, Carmen Coronado, María-Jesús Movilla-Fernández

**Affiliations:** 1 Facultade de Enfermaría e Podoloxía, Universidade da Coruña, Campus de Esteiro, CP: 15403 Ferrol, Spain; 2 Laboratorio de Investigación Cualitativa en Ciencias da Saúde (CCSS), Grupo de Investigación Cardiovascular (GRINCAR), Universidade da Coruña, Ferrol, Spain; 3 Bloomberg Faculty of Nursing, University of Toronto, Toronto, Canada; 4 Centre for Critical Qualitative Health Research (CQ), University of Toronto, Toronto, Canada

**Keywords:** Cervical intraepithelial neoplasia, Papillomavirus infections, Health education, Patient care, Patient satisfaction, Patient rights

## Abstract

**Background:**

Internationally, women with cervical intraepithelial neoplasia (CIN) lack knowledge about their disease, which limits their ability to take responsibility for self-care and creates negative psychosocial effects, including marital problems. Normally, screening is performed in primary care, and in case of abnormal results, the patient is referred to specialized care for follow-up and treatment. Given the lack of international literature regarding patients’ experiences in primary and specialized healthcare, our study aims to: (a) investigate how women with CIN perceive the communication and management of information by healthcare providers at different moments of their healthcare and (b) identify these women’s informational needs.

**Methods:**

A qualitative exploratory study was carried out in a gynecology unit of a public hospital of the Galician Health Care Service (Spain). Participants were selected through purposive sampling. The sample consisted of 21 women aged 21 to 52 years old with a confirmed diagnosis of CIN. Semistructured interviews were recorded and transcribed. A thematic analysis was carried out, including triangulation of researchers for analysis verification.

**Results:**

Two analytical themes were identified. The first was communication gaps in the diagnosis and management of information in primary and specialized healthcare. These gaps occurred in the following moments of the healthcare process: (a) cervical cancer screening in primary care, (b) waiting time until referral to specialized care, (c) first consultation in specialized care, and (d) after consultation in specialized care. The second theme was participants’ unmatched informational needs. The doubts and informational needs of women during their healthcare process related to the following subthemes: (a) HPV transmission, (b) HPV infection symptoms and consequences, and (c) CIN treatment and follow-up.

**Conclusions:**

This study shows that women who have a diagnosis of CIN experience important healthcare informational challenges when accessing primary and specialized care that have several implications for their wellbeing. The information given is limited, which makes it difficult for women to understand and participate in the decision making regarding the prevention and treatment of CIN. Service coordination among different levels of care and the availability of educational materials at any given time would improve the patients’ healthcare experience.

## Background

Human papillomavirus (HPV) infection is one of the most common sexually transmitted infections worldwide [1] and is the cause of all cervical cancer cases [2]. Presently, the prevention of cervical cancer is based on vaccination against HPV infection and screening for the early detection of precancerous cervical lesions, known as cervical intraepithelial neoplasia (CIN) [1].

Internationally, researchers have shown that health care informational challenges for women diagnosed with CIN limit their ability to self-care [3, 4] and lead to several negative psychological effects [5, 6]. Women with CIN lack knowledge about their condition [3, 5, 7, 8] and experience anxiety [3, 9], fear of cancer [3, 10, 11], guilt, shame and feelings of stigmatization [12], and they also have problems in their social [13, 14] and intimate relationships [11, 14]. Researchers have also shown that there is no adequate flow of information between health care providers and patients [7, 9, 11] and that health care providers have knowledge gaps about infection, testing and HPV vaccination [15]. However, in Spain, there are no studies on the users’ experience in this context. Previous Spanish studies have focused on cervical cancer screening evaluation [16], the prevalence of precancerous lesions and the types of HPV present in cytological samples [17–19] as well as HPV vaccination [20–24].

Similar to that in many other countries, the Spanish National Health Care System [25] establishes that cytological screening should be performed in primary care, and in case of abnormal results, the patient should be referred to specialized care for follow-up and treatment. Given the lack of international and Spanish literature regarding patients’ experiences in primary and specialized health care, we have designed a study to (a) investigate how women with CIN perceive the communication and management of information by health care providers at different moments of their health care and (b) identify these women’s informational needs.

## Methods

A qualitative exploratory study was carried out in a gynecology unit in a public hospital of the Galician Health Care Service (Spain). A researcher (CFV) accompanied the gynecological team during the consultations and personally made the study known to the participants. Those who agreed to participate in the study were scheduled to meet at another time in a consultation room reserved for this research. In this meeting, participants were explained the objectives of the study and what their participation entailed. They were also given an information sheet, and the informed consent was read, clarified and signed before the interview.

Participants were selected through purposive sampling [26] fulfilling the following inclusion criteria: women between 21 and 65 years old, with diagnostic confirmation of CIN of any degree and able to communicate in Spanish. Women with a diagnosis of cervical cancer and physical and/or mental comorbidity that interfered with the description of the phenomenon were excluded from the study. Initially, 31 women agreed to participate, 5 of whom decided not to participate for personal reasons and another 5 of whom did not attend the interview without prior notice or justification. This resulted in a final sample of 21 participants: 15 were in preventive follow-up, and 6 were in follow-up after conization. The sociodemographic characteristics of the sample are described in Table 1.

**Table 1:** Sociodemographic characteristics

Participant	Age	Academic level	Marital status	No. Children	Type of diagnostic	Year of diagnostic
I-1	33	Graduate degree	Single (with partner)	0	CIN 1	2013
I-2	23	Compulsory secondary education	Single (with partner)	0	CIN 2	2015
I-3	29	Higher vocational training	Single (without partner)	0	CIN 1	2008
I-4	46	Graduate degree	Married	2	CIN 1	2015
I-5	25	Graduate degree	Single (with partner)	0	CIN 3	2015
I-6	33	Baccalaureate degree	Single (with partner)	0	CIN 3	2013
I-7	21	Associate degree	Single (with partner)	0	CIN 1	2012
I-8	26	Graduate degree	Single (with partner)	0	CIN 1	2014
I-9	45	Compulsory secondary education	Married	2	CIN 1	2014
I-10	42	Graduate degree	Married	1	CIN 3	2010
I-11	39	Higher vocational training	Separated (with partner)	1	CIN 2	2012
I-12	27	Graduate degree	Single (with partner)	0	CIN 2	2014
I-13	35	Higher vocational training	Married	1	CIN 1	2015
I-14	52	Graduate degree	Married	1	CIN 1	2010
I-15	25	Higher vocational training	Single (without partner)	0	CIN 1	2012
I-16	37	Associate degree	Single (with partner)	0	CIN 1	2007
I-17	29	Graduate degree	Married	0	CIN 1	2013
I-18	26	Graduate degree	Married	0	CIN 1	2010
I-19	48	Associate degree	Divorced (with partner)	1	CIN 1	2015
I-20	44	Baccalaureate degree	Married	2	CIN 3	2005
I-21	34	Higher vocational training	Single (with partner)	0	CIN 1	2014

The first author (CFV) conducted all semistructured interviews. The interview guide was based on the literature review and on the advice of three expert reviewers, two in qualitative methodology (MJMF, CC) and one in HPV infection (Table 2). The interviews were conducted from October to December 2015, with the majority of interviews lasting approximately 40 min. They were audio recorded and transcribed; after the verification of the accuracy of the transcription, recordings were destroyed. Field notes were integrated into the transcripts to enrich data.

**Table 2:** Semistructured interview script

Experience of being told the diagnosis by a health care professional.	
Management of information related to the diagnosis: HPV transmission, treatment, changes in lifestyle, etc.	
Experience of the process of receiving and searching for information.	
Information sources.	
Description of advice given by health professionals.	
Considerations to improve the information process based on their lives and experiences.	

The study obtained the approval of the Autonomic Committee of Research Ethics of Galicia (Spain) with registration code 2015/230 and had the permission of access to the field by hospital management. The interviewer (CFV), a nurse, did not belong to the gynecological service where the study took place. This allowed women to talk freely about their perceptions, without feeling that their participation would interfere with their health care. The interviewees had many doubts about the HPV diagnostic. The researcher addressed their informational needs at the end of each interview.

A thematic analysis was carried out [27], including identification of units of meaning and codes that were grouped into subcategories and analytical categories. Saturation was reached for the two themes presented in this article. Throughout this inductive process, analytical memos were developed to guide the analysis and ATLAS.ti was used for data management (version 7.5.10).

To ensure trustworthiness [28], triangulation of sources was carried out among three researchers (CFV, MJMF and DG) who read and analyzed the transcripts. The final categories were agreed upon by the entire team. The verification of information was carried out during the interviews, since it was not possible to meet participants again.

## Results

Two analytical themes were identified. The first theme was communication gaps in the diagnosis and management of information in primary and specialized health care. These gaps occurred in the following moments of the health care process: (a) cervical cancer screening in primary care, (b) waiting time until referral to specialized care, (c) first consultation in specialized care, and (d) after consultation in specialized care. The second theme was participants’ unmatched informational needs. The doubts and informational needs of women during their health care process related to the following subthemes: (a) HPV transmission, (b) HPV infection symptoms and consequences, and (c) CIN treatment and follow-up.

### Communication gaps in the diagnosis and management of information in primary and specialized health care

**Fig. 1:**
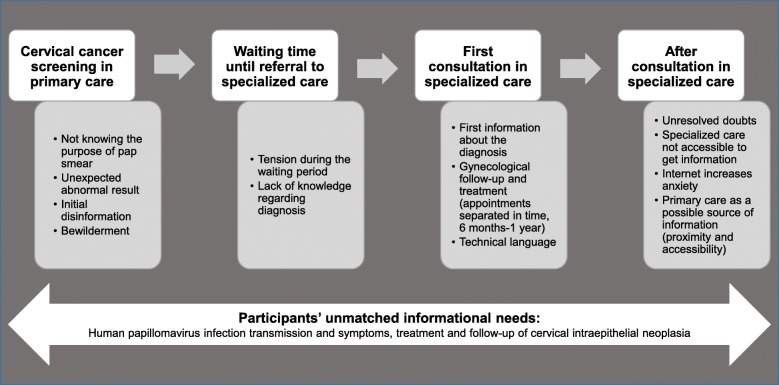
Experiences of women with CIN in relation to health care providers’ communication and management of information during the health care process at two levels of care: primary and specialized

Figure 1 presents a summary of the experiences of women with CIN in relation to health care providers’ communication and management of information during the health care process at two levels of care: primary and specialized. Four key moments were identified: cervical cancer screening in primary care, waiting time until referral to specialized care, first consultation in specialized care and after consultation in specialized care.

#### Cervical cancer screening in primary care

Several participants of this study expressed that they had pap smears performed without knowing their purpose. This led to perplexity when participants received an unexpected result of abnormal findings, and especially when the information was communicated by telephone.

*“You take this test to see if everything is all right, but what exactly? In the end, you don’t know what’s going on. You know that this test should be done, but what is it all about? What are they looking for? You don’t know.”* I-11

*“I was working when I got a phone call. I was told ‘the test showed some… atypical… cells’. It scared me to death. I burst out crying… I had no idea what it all meant.”* I-13

#### Waiting time until referral to specialized care

According to the participants’ accounts, some health care providers did not give information about the diagnosis, and limited themselves to referring women to a gynecological service. This became a situation of great uncertainty (which varied depending on the CIN severity) as the participants had to wait until an appointment to learn more about the diagnosis.

*“You are only told that you have a problem but without an explanation. You are absolutely confused. You ask for a gynecological appointment, but there’s a waiting period which only adds more anxiety. When you are told you have a problem, you want to know exactly what’s going on right away.”* I-4

#### First consultation in specialized care

Most women agreed that the first information they received came from gynecologists in specialized health care during the first consultation.

*“I came to the hospital for the first time, and it was there when the gynecologist asked me openly: ‘Tell me what doubts you have’. But, the thing is that I didn’t even have any doubts because I was completely ignorant about the subject… I only knew what the family physician had told me which was that I couldn’t ignore these results and that I had to be checked again…”* I-18

Under specialized health care, participants received information at the time of the scheduled consultations (every 6 months or once a year), and it was difficult or impossible for them to request an additional gynecological appointment to resolve their doubts.

*“You get some raw information, and later, when days go by, you start coming up with questions. Obviously, you cannot come [to specialized health care] every day to ask questions. They do try to explain things. I was given some good explanations [in specialized health care].… The thing is that afterwards you can’t stop thinking about all these things… but… you know, it’s not like you can come to ask questions every day.”* I-11

#### After consultation in specialized care

In search of additional information, our participants turned to the Internet but could not find answers to many of their questions, which generated more worries.

*“You are driven by fear, and it makes you look things up on the Internet, which is obviously not the best place. I learned this the hard way because it only made me feel more scared.”* I-1

Women pointed out that the solution could be that primary care providers are made responsible for information during their routine consultations. They identified primary care as the preferred level of care to obtain information on a continual basis due to the greater accessibility to health care professionals and availability of appointments.

*“Doubts keep coming up as time passes, but you feel you have nobody to speak to. You can’t call your gynecologist directly. However, you can go to your family physician. For example, there are phone consultations available, as well as face to face. You can ask questions this way.”* I-21

*“I think [the information given by professionals] is highly technical. I remember feeling much more relaxed after speaking to my family physician. Family physicians make an effort to explain it in basic terms so that you can understand it better.”* I-6

In summary, participants identified that their lack of previous information, the time when information was offered, the technical language used by specialists, and the lack of opportunity to ask questions outside the annual or semiannual appointment were circumstances that were interrelated and interfered with their understanding of CIN and its medical follow-up process.

### Participants’ unmatched informational needs

The relationship between CIN and cancer added tension and uncertainty to the follow-up process as illustrated by the following account of a participant: *“It might be cancer... All those things they don’t tell you, they don’t explain these things to you clearly”* (I-7). The participants’ unmatched informational needs included doubts about all key aspects of CIN and HPV infection, including transmission, symptoms, treatment and follow-up, as described below.

#### HPV transmission

The majority of the women said they knew that HPV is a sexually transmitted virus, although some questioned whether it could be “produced” by the woman/man’s own body.

*“Some people say that women produce the virus. Others tell you that’s not the case, that it comes from men that either produce it or pass it on. While others assure you that women can develop it either by producing it or getting it, like a yeast infection. I don’t know.”* I-11

Participants did not know how the infection was transmitted through distinct sexual practices, and wondered especially about possible transmission through oral sex.

*“I don’t know if you can get this simply by touching hands or something like that. Is it only transmitted through penetration or ejaculation…? You try to follow all necessary precautions, but you are not given more information.”* I-3

*“I have also read things…that the virus doesn’t only affect the cervix. Reportedly, Catherine Z. Jones’ husband had throat cancer caused by this. I don’t know whether it’s true or not. When you go to see the gynecologist, they only tell you about what you have down there [in the cervix]. They don’t tell you to be careful—that this or that can happen if you do certain sexual practices.”* I-15

Participants also wondered whether having acquired the infection could be due to infidelity on the part of their current partner, unaware of the fact that the virus has a long latency period.

*“I knew who I had been with. You have to ask the other person, though. You corner him and ask: ‘Who have you been with? Where?’ If I got CIN 1 in 2014, and in 2013 I got a negative result. You wonder… something must have happened in between. Supposedly, the pap smear was done right in the past. So, there wasn’t any doubts about the previous year. My reasoning was: if my conscience is clear, and you know that it wasn’t your doing, you automatically blame the other person.”* I-9

*“I had sex with two men…and that was it. I don’t really know if it is something that they pass on to you or if it develops in time…. I honestly don’t know.”* I-18

Some participants also considered other possibilities for transmission; they wondered if HPV could be acquired in public restrooms, sharing objects or by having previously been vaccinated against it.

*“You use your sponge in the shower, your partner has another one. But who knows? They are touching one another. If this is the case, it is obvious you are going to be exposed sooner or later. I don’t know if it is so easy for it to spread, only by touching infected objects.”* I-21

*“Or apart from sexual transmission, what about public restrooms? Could it be transmitted this way?”* I-18

*“I didn’t meet any of the risk factors: I wasn’t a smoker, I wasn’t taking any oral contraceptives, I hadn’t been with different partners so, I thought: ‘Why me? Was it the vaccine?’ I stopped asking the gynecologist, but surely I did used to ask this question every time I went to a consultation. According to them, there are many different virus strains, and I’m supposed to trust them. But, the truth is that I didn’t have anything, and then, suddenly, I got a positive result after getting the vaccine.”* I-10

#### HPV infection symptoms and consequences

When the participants were diagnosed with CIN, they asked themselves what it was they had, potential symptoms (as they did not feel anything unusual), where the lesions were located, and if it could affect other areas of the body.

*“What did I have down there? What was that? I don’t know. Maybe a little wart, a small injury.… What exactly? Abnormal cells of what?”* I-17

*“I can’t tell you where my lesions are exactly. You are not told that information. Only that you have CIN 1, and that’s it.”* I-16

*“I really don’t know if the fact that my vagina gets swollen more on the inside than on the outside, if that has anything to do with the virus. I don’t think so. Sometimes I say to myself: Damn it. It is really swollen on the inside, it hurts, or whatever.”* I-18

*“Also, what other areas can be affected? Other than your cervix, … maybe it can spread to other parts of the body or whatever.”* I-15

Participants also lacked knowledge about the evolution of their precancerous lesions to cancer and the influence of the infection on fertility and pregnancy (contagion to the fetus, abortion or increased possibility of developing cancer), which also created reasons for concern.

*“So, I was like…. How fast can this develop? I went home thinking that I was bound to have cancer sooner or later.”* I-18

*“But, if it is not healed, can you have children? I’m not having them… not until I am completely cured at least. But, I have this doubt. Can you get pregnant if you’re not completely cured?”* I-3

*“Nobody told me, not even the gynecologist. I didn’t ask her; maybe she didn’t realize. But, she could have told me: ‘Look, you can’t pass it on to the fetus, or there’s a risk of it developing or not, or for it to stay stable. However, if it develops, you may have to get a test that can lead to miscarriage or not.’ Nobody told me that.”* I-21

Finally, participants had doubts about how the virus could affect their partners, if they needed medical follow-up or if they needed to get tested for HPV infection.

*“We are a couple. I mean, I might be hurting him. I got the impression that I had to think that this was a private matter and that it wouldn’t affect anybody else. I think otherwise.”* I-1

*“There should be a way for men to have it checked. Make them have a study or test just to see if they are carriers. That would be really important.”* I-9

#### CIN treatment and follow-up

Our participants showed confusion about the meaning of the lab results carried out during the gynecological follow-up and had difficulty understanding the technical language used by health care professionals.

*“The word ASCUS [atypical squamous cells of undetermined significance] comes to mind. It has shown up several times in my pap test. I hear it in isolation in the conversation with my gynecologist and I think, what’s ASCUS? What’s the relationship of ASCUS with all this? It’s a long speech I’m given with a thousand words I don’t understand.… I don’t even know where to begin to ask questions.”* I-18

*“The doubt that I had was the number that [the gynecologist] told me… 16 or 18… I can’t remember the number she said exactly now. I know that it was important because I read it. I came home, and read it one more time. I had no idea what it meant. The whole thing of the number of the virus left me with many doubts.”* I-13

They did not understand the decisions made by gynecologists during treatment and follow-up either. For instance, the reasons were unclear for delaying conization once lesions were detected or for not being given any treatment.

*“I was mentally prepared for the gynecologist telling me that I had to undergo the conization And then, it was like why are you not doing it? How come I only need the vaccine? Don’t you want to treat me? Do you want this to get worse? My fear was ‘is this going to get worse’? CIN 2 means that it may be cervical cancer?”* I-2

They were also doubtful about the usefulness of recommendations such as vaccination, condom use and smoking cessation.

*“People say that even with condom use… with just the minimal touch… it can happen. The condom is not really that effective because it doesn’t cover the whole area. I don’t know...”* I-19

*“And I was told after the diagnosis that it was beneficial for me to get vaccinated. I didn’t understand why. If I had already had sexual relationships, why would I need the vaccine? I was told to get the vaccine anyway.”* I-17

*“I knew that tobacco had a link to bladder cancer and this type of cancer, but I didn’t relate one thing to the other [cervical cancer and tobacco]. I didn’t think I had to take any special measures regarding tobacco.”* I-14

Additionally, they considered if there were factors that determine whether a person is prone to developing cervical cancer, such as the influence of mood or genetics.

*“There might be people who are more predisposed to get this. I don’t know if it depends on whether your defenses are low.”* I-21

*“The conization coincided with a time when I was feeling low because my father had died. So, I asked the gynecologist at one of the consultations if it could have an effect on all this. The two times I was feeling emotionally down was when the test came out positive.”* I-10

*“Plus, in my family, there is a genetic predisposition [to cancer], and you know that, sooner or later, it’s going to be your turn.”* I-16

The information they received did not allow them to differentiate between having an HPV infection and having CIN. They considered that if they had been treated for CIN by surgical intervention, the virus would disappear and they would be “cured” without the possibility of recurrence.

*“Now that I am sort of discharged from the consultations, I don’t know whether I can infect other people. In theory, I had all my cells removed [conization done]. Supposedly, I don’t have anything anymore, do I?”* I-6

*“I underwent surgery, and it was a success. I came to have regular pap smears and everything was OK. I didn’t know that it could happen again. I had this 10-11 years ago, and now it’s here again. Why?”* I-20

## Discussion

Our findings coincide with the international literature in that women with CIN showed a lack of knowledge about their condition [3, 5, 7, 8] and that the communication between health care providers and patients was limited [7, 9, 11]. Women found it difficult to resolve doubts [7], which generated feelings of fear and angst [11] and long periods of great concern between consultations [6]. We concur that an abnormal pap smear result is unsettling in part due to a previous lack of information regarding its purpose [6, 9]. Receiving this result over the phone produced negative feelings because participants did not have the possibility to ask questions [13, 29], causing them to resort to looking for information on the Internet, a source that did not solve their concerns and, for some, generated more fear [7, 11, 30, 31].

Our study is also a pioneer in identifying different moments in the health care process, from the diagnosis of CIN in primary care to medical follow-up and treatment in specialized care, in which women experience health care informational challenges. Another novel fact is that women in our study emphasized that the information provided by their primary care providers at the time of the diagnosis was scarce or null, and that family physicians/midwives limited themselves to referring participants to the gynecology service. Such a way to proceed led to concern and fear during the waiting period (from days to weeks) due to the lack of accurate, accessible information about their situation. Participants expressed the need for reaching out to a health care professional to access information at any point and considered primary care providers as the best option.

Our participants shared the same doubts about key information regarding HPV infection and CIN as participants of international studies. Most participants were aware that HPV infection was acquired through sexual intercourse, although there were exceptions. Some of them thought that the body could produce the virus or, as Marlow et al. [32] showed in their study, some of our participants wondered if the infection could be acquired in other ways (e.g., sharing objects or being vaccinated against HPV). As previously described, at the time of diagnosis, participants did not know that the virus has a long latency period [32]. The lack of information has had psychological and social impacts on their lives because it raised questions about their partners’ fidelity [7, 11].

Similar to other studies, our participants did not know exactly what it meant to have CIN [30], if it produces symptoms [32, 33] and how it might progress to cancer [9, 11, 13, 30]. They especially wondered if there were factors that could stimulate the development of cervical cancer, such as genetic factors or psychological disposition. As described by other authors, our participants considered whether the infection could affect areas other than the cervix [34, 35] and could affect their fertility [9, 30, 36], as well as what effects it could have during pregnancy [7, 30] and for their partners’ health [30]. Our study also confirms previous findings that women have difficulty understanding the language used by health care providers [30], as well as the meaning of test results [6, 9], the decisions that providers make and the recommendations they offer during treatment and follow-up [3, 5, 34, 37].

Finally, our study reveals that a consequence of the lack of information about HPV transmission through distinct sexual practices (e.g., oral sex) was that participants were living with uncertainty, which interfered with their sexuality, as they did not know adequate preventive practices for themselves and their partners. Such findings show that in our interviews sexuality was discussed in a comprehensive way but did not occur in consultations with health care professionals; couples’ sexual practices in the context of prevention were not addressed. In addition, participants’ lack of understanding made it impossible for them to differentiate between HPV infection and CIN. Some of them did not understand why they were not subjected to conization to treat CIN. They considered that such a treatment would eliminate the virus and they would be cured. Based on this belief, it was difficult for them to understand the gynecologists’ decision to recommend “only” medical follow-up for their precancerous lesions and made them feel they were receiving substandard health care.

### Strengths and limitations

We believe that two factors greatly contributed to the success of this study. First, the interviewer (CFV) did not belong to the gynecological service where the study was conducted, which allowed participants to speak freely about their perceptions. Second, participants were informed that the study aim was to improve health care for women with CIN, which invited them to reflect and offer critique to the health care system in a positive way.

There were challenges for this qualitative study as well. A few participants had no familiarity with in-depth interviews and wanted to provide short, precise answers or were pressured for time (two interviews lasted 11 and 13 min); however, the majority of them took the time to provide detailed accounts of their experiences. In addition, this study had a heterogeneous sample regarding age and education because of its exploratory purpose. Future studies should further investigate the specific needs of different groups of users of the national health care system, such as young women or immigrants, and evaluate the best way to offer information to each group.

## Conclusions

This study shows that women who have a CIN diagnostic experience undergo important health care informational challenges when accessing primary and specialized care, which have several implications for their wellbeing. Participants described informational needs for HPV transmission, symptoms, treatment and follow-up of their condition. The information that women receive is limited, which makes it difficult for them to understand and participate in decision making regarding prevention and treatment.

We propose that health care providers should take into account the different moments of the process when providing information to patients, as needs differ from the time of cervical screening in primary care to CIN follow-up and treatment in specialized care. Within the context of a publicly funded national health care system, primary care providers (family physicians and midwives) have been identified by our participants as preferred professionals to provide information, given their accessibility and for their on-going relationship with them. In specialized health care, sparse appointments make the information process fragmented, and health care providers’ technical language are barriers to overcome.

To facilitate an effective exchange of information with patients, we believe that health care managers in primary and specialized care levels should create opportunities to meet users’ informational needs and take into account the informational needs manifested by women in our study to guide future interventions. Health care users could also greatly benefit from access to accurate information in services and online. This study proposes either the identification of reliable sources of information already available or the development of educational materials (digital and printed) to be shared among all levels of health care to support health education in clinical settings and timely access to accurate information between appointments.

## Data Availability

There is an ethical and legal restriction on sharing our data. According to the Autonomous Committee of Research Ethics of Galicia (Spain) only the researchers can have access to the data.

## Abbreviations

ASCUS: Atypical squamous cells of undetermined significance
CIN: Cervical intraepithelial neoplasia
HPV: Human papillomavirus
